# The lucent yet opaque challenge of regulating artificial intelligence in radiology

**DOI:** 10.1038/s41746-024-01071-2

**Published:** 2024-03-15

**Authors:** James M. Hillis, Jacob J. Visser, Edward R. Scheffer Cliff, Kelly van der Geest – Aspers, Bernardo C. Bizzo, Keith J. Dreyer, Jeremias Adams-Prassl, Katherine P. Andriole

**Affiliations:** 1 Data Science Office, Mass General Brigham, Boston, MA USA; 2 Department of Neurology, Massachusetts General Hospital, Boston, MA USA; 3 Harvard Medical School, Boston, MA USA; 4 Department of Radiology & Nuclear Medicine, Erasmus Medical Center, Rotterdam, The Netherlands; 5 Program on Regulation, Therapeutics and Law, Brigham and Women’s Hospital, Boston, MA USA; 6 Department of Medical Technology, Erasmus Medical Center, Rotterdam, The Netherlands; 7 Department of Radiology, Massachusetts General Hospital, Boston, MA USA; 8 Faculty of Law, University of Oxford, Oxford, UK; 9 Department of Radiology, Brigham and Women’s Hospital, Boston, MA USA

**Keywords:** Health policy, Medical imaging

## Introduction

The potential applications of artificial intelligence and machine learning (AI/ML) in medicine are progressing rapidly. AI is a broad term that refers to the intelligence of computer and software systems, while ML is a type of AI involving computers learning through pattern recognition methods including artificial neural networks. Radiology is a frontrunner in this space: between 2015 and early 2020, 129 radiology AI/ML devices received regulatory clearance from the United States Food and Drug Administration (FDA), and 126 devices received the *Conformité Européenne* (CE) mark in Europe^1^. These approvals are only accelerating, with the FDA clearing 126 radiology AI/ML devices in the twelve months to July 2022^2^. Both the speed and volume of AI/ML devices present a delicate balance for regulatory bodies: ensuring the safety and effectiveness of devices while keeping pace with the clinical innovation and value that they may provide.

Here we discuss the current and future regulatory landscapes of AI/ML in radiology, and we highlight pressing challenges that are critical for regulatory bodies to traverse. Other medical specialties will soon face similar hurdles as AI/ML become increasingly ubiquitous and may benefit from considering these challenges proactively.

## Current regulatory landscape

Like other medical regulations, those for radiology AI/ML use a risk-based approach that considers safety and effectiveness. This approach is heterogeneous between jurisdictions (Table 1), with the points of difference reflecting many of the most challenging areas to regulate.

**Table 1:** Summary of radiology AI/ML device regulatory approaches in different jurisdictions

Regulatory agency	United States Food and Drug Administration	European Union Medical Device Regulation
Organizational structure for regulatory clearance	Centralized process through the Center for Devices and Radiological Health	Decentralized process through ~40 Notified Bodies
Typical risk classification for radiology AI/ML device	Class II	Class IIa
Predicate pathway	510(k) pathway (for substantially equivalent device to predicate) for Class I/II device.	No predicate pathway.
Product types / codes	Regulation numbers define the de novo / 510(k) pathways, including the associated ‘special controls’; each regulation number may then have multiple product codes within it; there are 26 different product codes that have been used for radiology AI/ML devices^2^.	All software is defined by the code MDA0315; this code defines which Notified Body can be designated to evaluate a device.
Minimal device metrics	Defined for some product codes (e.g., QFM for radiological computer-assisted prioritization software for lesions requires area under curve >0.95). Many product codes do not have a defined minimal metric.	No defined minimal metrics. The metrics are linked to the device claims.
Model change process	Draft guidance to define changes as part of the Predetermined Change Control Plan. Separate guidance for deciding when to submit a 510(k) for a software change to an existing device.	Guided by whether a change is considered substantial. Predetermined plans are not part of the MDR.
Accelerated / conditional pathway	No; the Breakthrough Devices Program facilitates prioritized review but still requires the applicable regulatory review.	No
Consideration of cost-effectiveness in clearance	No	No
Post-market surveillance	Yes	Yes
Database of approved devices	FDA website^2^	EUDAMED database^10^

The US FDA regulates medical software as devices, as defined in section 201 of the Food Drug and Cosmetic Act^3^, and has considered most radiology AI/ML devices as Class II^2^. Class II classification indicates that a device is moderate-risk and requires ‘special controls’ that are specific to the device to assure safety and effectiveness. A novel device is granted a de novo request that identifies these special controls, while a less burdensome 510(k) request allows clearance of subsequent devices considered substantially equivalent to the ‘predicate’ device. This process differs from the more intensive premarket approval process that is used for high-risk Class III devices^4^. As an example of the de novo / 510(k) pathways, Viz.ai was granted a de novo request for an acute stroke large vessel occlusion (LVO) detection device in February 2018 under the newly created regulation number 892.2080 with the designated QAS product code^5^. This device is considered radiological computer-aided triage and notification software, and one of the special controls involves demonstrating how the device will provide effective triage. By July 2022, 30 devices had received subsequent 510(k) clearance under the QAS product code; a related product code of QFM was also created under regulation number 892.2080, with the first device using the Viz.ai device as a predicate^2,6^.

The European Union (EU) Medical Device Regulation 2017/745 (MDR) regulates medical software as active devices, as defined in Article 2 of the MDR^7^. Unlike the centralized FDA approval process, the EU market approval (CE mark) process for devices occurs in a decentralized manner through one of ~40 Notified Bodies. This approval then covers market access for the whole EU. The classification is guided by Rule 11 in Annex VIII of the MDR, which is focused on the intended purpose of the device and states that “software intended to provide information which is used to make decisions with diagnosis or therapeutic purposes” are Class IIa devices. There are exceptions, including designating devices as Class IIb or III if such decisions may cause either a serious deterioration in health or death or when the software monitors vital physiological parameters in certain situations. Devices are then considered for approval within this classification; there is no further breakdown to regulation numbers or product codes like the de novo and 510(k) request pathways with the FDA. While the EU has created a central database called the European Database on Medical Devices (EUDAMED), its full functionality has been delayed and mandatory use is not yet enforced^8^. The aforementioned Viz.ai LVO algorithm has received clearance as a device in the EU^9^; while EUDAMED lists Viz.ai as a manufacturer, it does not yet list them as having any devices^10^.

A benefit of having the de novo and 510(k) pathways is that the burden of the regulatory approval process can vary based on the incremental risk of a device compared to other available devices. Manufacturers can also take advantage of clearer expectations of device features, including performance metrics from predicate devices. The 510(k) pathway has, however, been criticized for the divergence in the AI/ML tasks performed by devices and their predicates^11,12^. At a broader level, 510(k) devices are the most recalled medical devices, which has raised concerns about the pathway; it is also possible to use a predicate device that has been recalled, with descendent devices having a higher risk of their own recall^13,14^. In addition, the less burdensome nature of the 510(k) pathway may cause manufacturers to opt for 510(k) approval over a de novo approach, potentially leading them to curtail innovative features that extend beyond a predicate device.

**Fig. 1:**
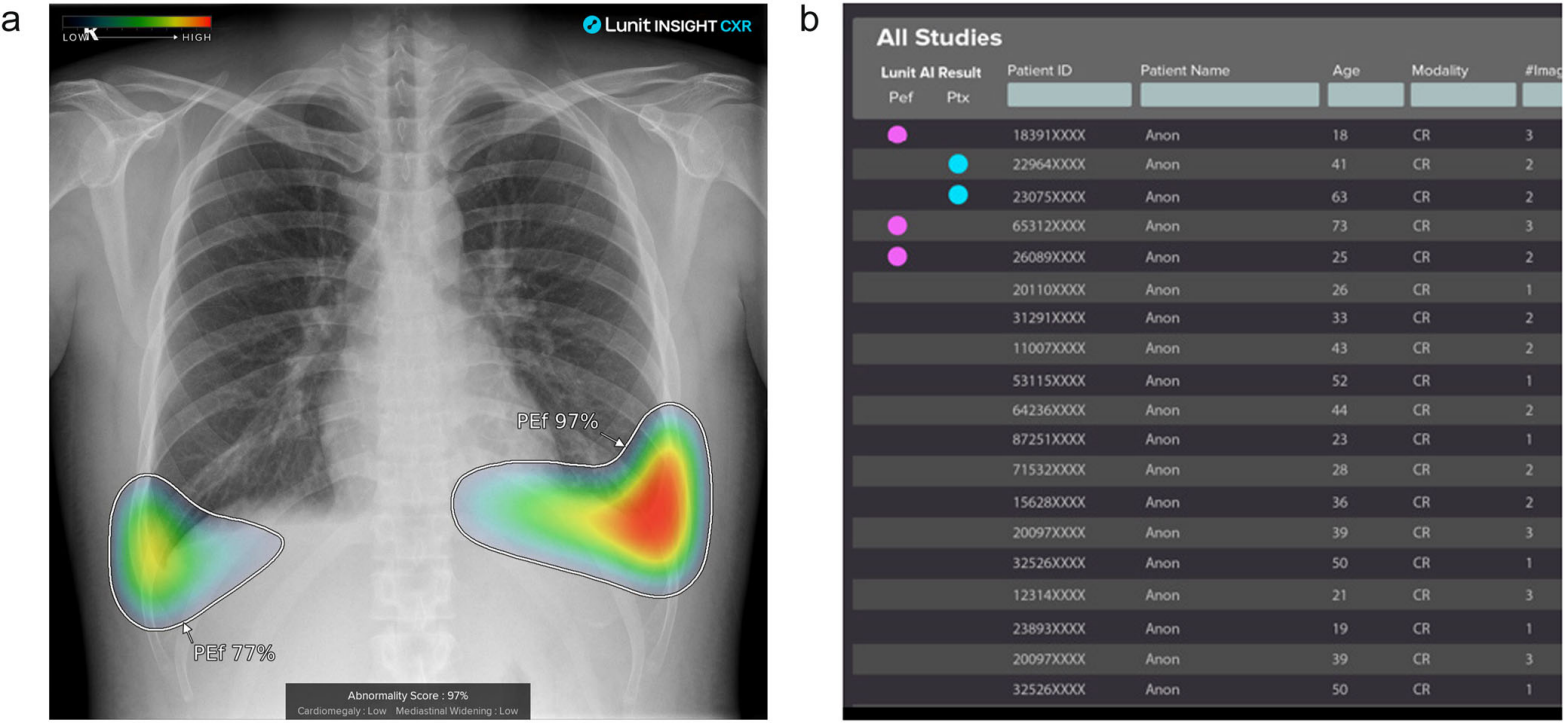
Comparison of how the same AI algorithm can be cleared as different devices in different regulatory jurisdictions. The same pleural effusion detection algorithm as a CE-marked device in the European Union (**a**) and US FDA-cleared triage device (**b**). While the manufacturer defines the intended purpose and clinical claims of a device and, therefore, chooses the regulatory approach, these decisions can be driven by the regulatory framework in each jurisdiction. The CE-marked device provides a heatmap of the region of interest. In contrast, the FDA-cleared device, which is for triage under the QFM product code, only provides notification of which cases are positive for pleural effusion, as indicated by the pink dots. In addition, the CE-marked device is for 10 different findings, while the FDA cleared device is for only 2 findings (pleural effusion and pneumothorax). These images are taken from the manufacturer’s website^16^.

While the MDR may impart its own limitations on a device, its processes typically allow a manufacturer to obtain regulatory approval for broader features in a less onerous manner than the FDA. This approach is exemplified in ‘comprehensive’ chest radiograph algorithms from Annalise.ai, Lunit, and Qure.ai. The CE-marked versions of these algorithms detect 124, 10, and 15 different chest radiographic findings, respectively^15–17^. In contrast, the FDA has cleared these same algorithms for just 5, 2, and 1 findings, respectively. Furthermore, while the Annalise.ai and Lunit FDA-cleared devices are limited to providing binary triage information (e.g., pleural effusion present or absent), the CE-marked versions of the devices can provide localization information such as heat maps (Fig. 1).

## Future regulatory landscape

As AI/ML devices increase in use and complexity, regulatory approaches will need to evolve rapidly to address these and further challenges. Recent developments in generative AI/ML, especially the release of the large multimodal model GPT-4 from OpenAI, underscore how quickly these advances can occur^18^.

The FDA released a discussion paper proposing a regulatory framework in 2019^19^, an action plan in 2021^20^, and draft guidance on Predetermined Change Control Plans (PCCPs) for device modifications in 2023^21^. These documents describe important areas, including good machine learning practice, algorithm bias and robustness, continuous learning (software that adapts incrementally over time), and assessment of real-world performance. The pace of innovation is clear, though none of these documents reference “generative AI” or “large language model” specifically. While the principles that they do reference will remain, there will be many additional considerations for generative AI, especially as possible device inputs and outputs increase from a limited set to a much larger (potentially infinite) set^22,23^. Separately the FDA has attempted to strengthen the 510(k) process more broadly, albeit further improvements have been proposed, including establishing more robust performance criteria and related testing methods^24^.

Medical uses of AI are also covered in the EU’s flagship regulatory proposal, the AI Act. Designed to both foster innovation and protect citizens’ fundamental rights, this proposed Act would distinguish between different risk categories, imposing regulations ranging from notification requirements to outright bans for each tier. While detailed provisions are still subject to amendment and intensive negotiations, Chapter 3 lays down the obligations of providers and users of ‘High-Risk AI Systems’. It builds on and is closely linked to existing product regulation approaches, including conformity assessments, under the ‘New Legislative Framework’^25^.

Key regulatory challenges remain and we consider several below. In doing so, we also consider the lessons that can be learned from parallels with pharmaceutical regulation.

### Enhancing post-market surveillance

The FDA and MDR approval processes for radiology AI/ML devices are mostly based on model performance with retrospective data. By nature, these data cannot encompass all situations a device may encounter following clearance, nor will they account for data drift (when a change in input data, such as a switch of MRI machines from 1.5 T to 3 T, decreases algorithm performance). Current post-market surveillance focuses on device malfunctions and serious injuries or deaths rather than maintaining ongoing device performance. Annex XIV of the MDR requires post-market clinical follow-up, although it provides flexibility for this requirement through the depth and extent being proportionate to the intended purpose and risks of a device^7^; for the most part, there has not been ongoing, systematic assessment of radiology AI/ML device performance. In contrast, post-approval clinical trials and real-world evidence, such as the FDA’s collaborative Sentinel system^26^, are both critical to pharmaceutical development and pharmacovigilance. They have revealed many safety events and even led to the withdrawal of drugs^27^. The benefits of the Viz.ai LVO detection device have started to be shown in real-world evidence, including its ability to decrease workflow times. However, these studies have spawned from academic and related purposes rather than regulatory requirements^28–30^.

### Supporting continuous/active learning

AI/ML devices can have an iterative ability to continue to learn, especially as more training data become available. Such updates may occur in a continuous, automatic manner through a model or in a discrete, manual manner with human input. The former involves greater risk and therefore warrants more regulatory attention, but the latter still involves more risk than most current models that have a closed system and are ‘frozen’. Strategies have been proposed to enable continuous learning, including retesting and simulated checks^31^. The recent draft guidance from the FDA on PCCPs also describes important components of device updates, including re-training practices and performance assessment^21^. However, it can be challenging to know the future changes that will be necessary at the time of submitting for regulatory clearance. To overcome this barrier, companies may try to make PCCPs more encompassing and less specific; it is not yet clear how this scenario will play out. If the regulatory burden of a PCCP submission is too great, manufacturers may forego a PCCP, similar to opting for the 510(k) pathway over a de novo approach.

### Enabling conditional clearances/approvals

Conditional clearances and approvals with appropriate regulatory guardrails could enable AI/ML devices that do not yet have sufficient evidence for a full assessment of safety and effectiveness to obtain this further evidence through post-clearance/approval studies. Accelerated approval pathways (called conditional marketing authorization in the EU) have been used for over 30 years to enable pharmaceuticals to reach patients faster based on preliminary evidence so that patients can benefit from drugs that are ‘reasonably likely’ to offer clinical benefit while further clinical trials are performed^32,33^. These pathways are not without their own challenges: many accelerated approvals have either not completed confirmatory trials or failed to verify their clinical benefit^32,34^. The coupling of post-market surveillance, continuous/active learning, and conditional clearances/approvals, which all require ongoing assessment of devices, provides an opportunity to be stricter than pharmaceutical approvals in ensuring completion of confirmatory studies. Such assessment could be particularly streamlined for radiologic AI/ML devices that have real-time feedback on device performance and accuracy.

### Moving beyond explainable and verifiable AI

Many current radiology AI/ML devices replicate tasks that radiologists could perform with the device providing benefit through a decrease in time to interpretation and/or time of interpretation. The outputs of these devices have therefore been verifiable, and the ‘thinking’ of the devices is explainable. As devices become more complex, especially when predicting a future clinical outcome, verifiability and explainability may become less clear. While this black-box nature can be instinctively unsettling for clinicians, it is analogous to the many medications that have been approved despite an incomplete understanding of their pharmacologic mechanism—including common medications such as paracetamol and lithium. A key enabler for this transition will be an increased focus on device performance on clinical outcomes rather than only model metrics^35^.

### Enabling autonomous AI/ML

A question arising across medical and non-medical industries is whether AI/ML devices can function autonomously. In radiology, this question entails myriad considerations, including regulatory limitations, medicolegal implications, and societal acceptance. There are already mooted use cases for autonomous AI/ML in radiology, including for ‘detecting normal’ (e.g., eliminating the need to interpret a chest radiograph that a ‘comprehensive’ algorithm has called normal). The developments in generative AI/ML further increase the possibilities of autonomy (e.g., an AI/ML device can more easily create a detailed radiology report). The regulatory approach will need to consider both when device autonomy is acceptable and how to ensure appropriate escalation back to a radiologist or other clinician when necessary.

## Conclusion

While it is lucent that regulations should ensure the safety and effectiveness of radiology AI/ML devices, the balance of keeping pace with AI/ML innovation makes the immediate next steps for regulatory evolution more opaque. We described many challenges that regulatory bodies will need to continue to consider as the potential of AI/ML in radiology and medicine more broadly is realized.
